# Pre-operative Machine Learning for Heart Transplant Patients Bridged with Temporary Mechanical Circulatory Support †

**DOI:** 10.3390/jcdd9090311

**Published:** 2022-09-19

**Authors:** Benjamin L. Shou, Devina Chatterjee, Joseph W. Russel, Alice L. Zhou, Isabella S. Florissi, Tabatha Lewis, Arjun Verma, Peyman Benharash, Chun Woo Choi

**Affiliations:** 1 Division of Cardiac Surgery, Heart and Vascular Institute, Department of Surgery, Johns Hopkins University School of Medicine, Baltimore, MD 21287, USA; 2 School of Medicine, University of Maryland, Baltimore, MD 21201, USA; 3 College of Letters & Science, University of California, Los Angeles, CA 90095, USA; 4 Division of Cardiac Surgery, David Geffen School of Medicine at UCLA, Los Angeles, CA 90095, USA; 5 Department of Cardiothoracic Surgery, Virtua Health, Virtua Our Lady of Lourdes Hospital, Camden, NJ 08103, USA

**Keywords:** machine learning, heart transplant, cardiac surgery, mechanical circulatory support

## Abstract

**Background:** Existing prediction models for post-transplant mortality in patients bridged to heart transplantation with temporary mechanical circulatory support (tMCS) perform poorly. A more reliable model would allow clinicians to provide better pre-operative risk assessment and develop more targeted therapies for high-risk patients. **Methods:** We identified adult patients in the United Network for Organ Sharing database undergoing isolated heart transplantation between 01/2009 and 12/2017 who were supported with tMCS at the time of transplant. We constructed a machine learning model using extreme gradient boosting (XGBoost) with a 70:30 train:test split to predict 1-year post-operative mortality. All pre-transplant variables available in the UNOS database were included to train the model. Shapley Additive Explanations was used to identify and interpret the most important features for XGBoost predictions. **Results:** A total of 1584 patients were included, with a median age of 56 (interquartile range: 46–62) and 74% male. Actual 1-year mortality was 12.1%. Out of 498 available variables, 43 were selected for the final model. The area under the receiver operator characteristics curve (AUC) for the XGBoost model was 0.71 (95% CI: 0.62–0.78). The most important variables predictive of 1-year mortality included recipient functional status, age, pulmonary capillary wedge pressure (PCWP), cardiac output, ECMO usage, and serum creatinine. **Conclusions:** An interpretable machine learning model trained on a large clinical database demonstrated good performance in predicting 1-year mortality for patients bridged to heart transplantation with tMCS. Machine learning may be used to enhance clinician judgement in the care of markedly high-risk transplant recipients.

## 1. Introduction

Orthotopic heart transplantation remains the gold standard therapy for patients with end-stage heart failure [1]. However, some patients on the waiting list can have urgent hemodynamic instability before a donor heart becomes available. In these cases, temporary mechanical circulatory support (tMCS) devices, including venoarterial extracorporeal membrane oxygenation (VA-ECMO), intra-aortic balloon pump (IABP), and percutaneous ventricular assist devices (PVAD), may be used as a bridge to transplant (BTT). In 2018, the United Network for Organ Sharing (UNOS) implemented a revised heart allocation system in which patients with uncomplicated, durable left ventricular assist devices (LVAD) saw a decrease in waitlist priority, while those supported with tMCS were bumped up to the highest priority [2].

Consequently, the use of tMCS as a bridge to transplant strategy has steadily increased [3], yet, existing pre-operative risk evaluation methods for determining post-transplant mortality have not been thoroughly validated in this distinctively complex and high-risk population [4,5,6,7,8]. For example, the Index for Mortality Prediction After Cardiac Transplantation (IMPACT) score [6] predicts 1-year post-transplant mortality. However, it was developed in a general heart transplant cohort and remains unvalidated for those bridged with tMCS. Similarly, the CARRS risk score [7], which is meant for a high-risk cohort, remains unvalidated in tMCS bridge patients and was developed using single-center data.

Machine learning (ML) has emerged as a powerful tool with wide biomedical applications. State-of-the-art ML algorithms which utilize supervised learning techniques can analyze complex relationships between inputted variables (“features”) to predict outcomes, often outperforming simpler models like regression [9,10]. These strategies have demonstrated great promise in important clinical issues such as asymptomatic cardiovascular disease risk [11], general heart transplant outcomes [12], and ischemia analysis from cardiac imaging [13]. Using a large national database, we sought to build an interpretable ML model which predicts 1-year post-transplant mortality for tMCS BTT patients.

## 2. Materials and Methods

This study was approved by the Johns Hopkins School of Medicine Institutional Review Board (IRB00159748) with a waiver of informed consent.

### 2.1. Patient Selection

**Figure 1:**
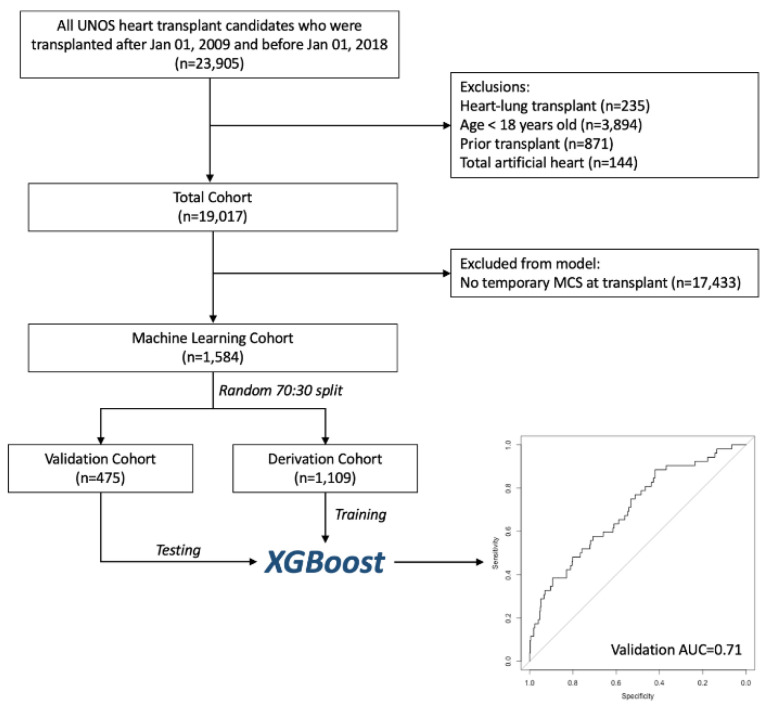
Flow diagram showing inclusion and exclusion criteria for this study’s cohort. UNOS: United Network for Organ Sharing; MCS: mechanical circulatory support.

We included all adult (≥18 years old at time of listing) orthotopic heart transplant recipients in the United Network for Organ Sharing (UNOS) database between 1 January 2009 and 31 December 2017. Re-transplant and multi-organ transplant patients, as well as those with a total artificial heart, were excluded (Figure 1). Patients belonged to the temporary mechanical circulatory support (tMCS) group if they were supported with venoarterial extracorporeal membrane oxygenation (VA-ECMO), intra-aortic balloon pump (IABP), or percutaneous ventricular assist device (PVAD) at the time of transplant.

### 2.2. Primary Outcome

Our primary outcome was 1-year post-transplant mortality.

### 2.3. Machine Learning Model Development

Our ML method of choice was extreme gradient boosting (XGBoost [14]). We used a 70:30 train:test split, meaning that 70% of patients were randomly selected as our development/training cohort and the remaining 30% were used as our validation/testing cohort, making sure to balance the number of events in both groups. Variables not able to be known at or before the time of transplantation (i.e., data collection for variable had to have taken place after the transplant procedure, such as post-operative complications) were manually identified and excluded. For variables which have more than one collection time point (e.g., at time of listing or at time of transplant), all time points were collected and appropriately labeled. Continuous or ordinal variables were treated numerically. Categorical variables were one-hot encoded. A grid search was used to optimize XGBoost hyperparameters and to prevent model overfitting. We tested the following hyperparameter combinations in the grid search: maximum tree depth [2, 3, 4], eta [0.5, 0.1, 0.01], gamma [0, 10, 15, 20], and minimum child weight [0, 10, 20]. Since our primary outcome class was highly imbalanced (i.e., many more survived compared to those who died), we attempted to control for this by setting the “scale_pos_weight” parameter to 7.8, which was the ratio of negative instances (“0”, alive) to positive instances (“1”, dead), in accordance with the original XGBoost documentation. The final hyperparameters used were: max depth = 2, eta = 0.01, gamma = 15, and minimum child weight = 10. We used a local model-agnostic method called Shapley Additive Explanations [15] (SHAP) to intuitively explain how the most salient features contributed to the model. It calculates scores (SHAP values) for each feature by comparing what the model predicts with and without that particular variable, thus measuring the incremental benefit of adding in any individual feature. Missing values were automatically imputed on a case-by-case basis in the XGBoost model, as described in the original documentation [14].

### 2.4. Statistical Analysis

Demographics and baseline patient characteristics were compared using Mann–Whitney U tests for continuous variables and Chi-square tests for categorical variables. Unadjusted 1-year post-transplant Kaplan–Meier survival curves were generated for the tMCS and non-tMCS groups and compared using the log-rank test. Bootstrapping with 1000 replications was used to assess model variability and to generate 95% confidence intervals (CIs). Area under the receiver operator characteristics curve (AUC), otherwise known as c-statistic, was the primary evaluation metric. Statistical comparisons for models were made using DeLong’s test [16]. A *p* value < 0.05 was considered statistically significant. Models were developed and statistical analyses were performed using R version 4.0.3, Python version 3.8.8, and Stata 17.

## 3. Results

### 3.1. Patient Characteristics

We identified a total of 19,017 patients who underwent isolated, first-time orthotopic heart transplantation (OHT) during the study period, out of which 1584 were bridged to transplant with tMCS (Table S1). IABP was used in 1190 cases, VA-ECMO in 114 cases, and PVAD in 356 cases. A total of 76 patients were supported on more than one type of tMCS device at the time of transplant. Among those bridged with tMCS, those who died within 1 year were more likely to be older (59 vs. 55 years old, *p* < 0.001), have diabetes (36% vs. 27%, *p* = 0.01), an implantable cardiac defibrillator (80% vs. 73%, *p* = 0.03), higher serum creatinine (1.30 vs. 1.14 mg/dL, *p* = 0.001), and a marginally higher body mass index (27.2 vs. 26.0 kg/m^2^, *p* = 0.02) (Table 1). Interestingly, those who died were more likely to have a lower pulmonary capillary wedge pressure (20.5 vs. 22.0 mmHg, *p* = 0.04) and less inotrope usage (50% vs. 58%, *p* = 0.04) at the time of transplant. Out of the 1584 tMCS patients, 1267 (70%) and 317 (30%) patients were randomly assigned to the development and validation cohorts, respectively. Compared to patients who did not require tMCS, the tMCS BTT group had lower survival at 1 year (87.9% vs. 90.3%, *p* = 0.006) (Figure S1).

### 3.2. Model Performance

**Figure 2:**
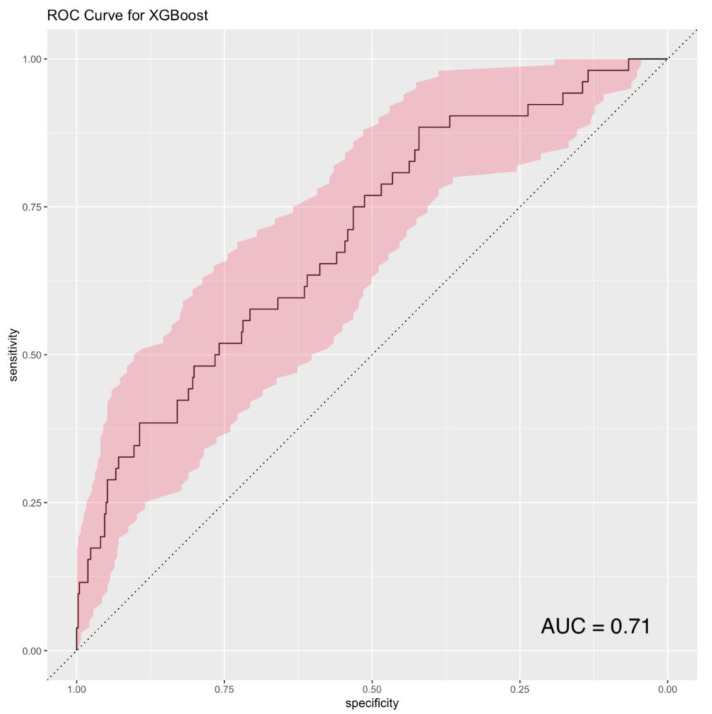
Receiver operator characteristics (ROC) curve for the XGBoost model. AUC: area under receiver operator characteristics curve.

Out of 498 pre-transplant variables available in the original dataset, 43 were automatically selected for inclusion in the final XGBoost model based on predictive utility (“gain”) (Table S2). The final XGBoost model performed favorably in predicting 1-year post-transplant mortality with an AUC of 0.71 (95% CI: 0.62–0.78) (Figure 2). The precision recall curve (AUCPR = 0.357) for the XGBoost model is provided in Figure S2.

### 3.3. Model Explanations

**Figure 3:**
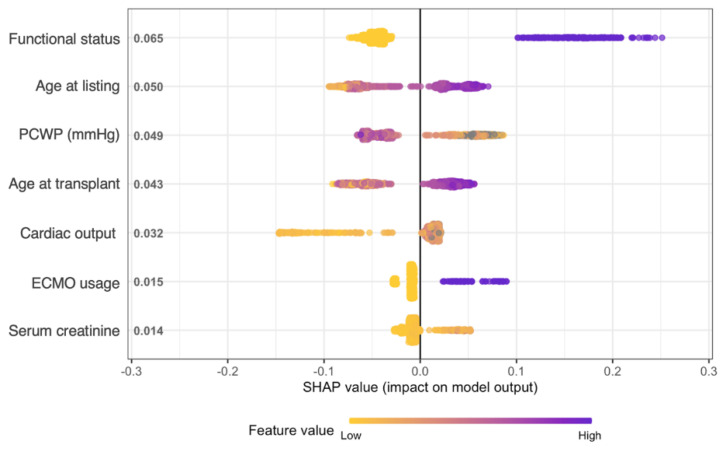
Shapley Additive Explanations (SHAP) summary plot for the top 7 most important features for model prediction, ranked by mean absolute SHAP value. Each dot represents one patient/observation. The *x*-axis is SHAP value, with a more negative value meaning that the feature for that observation drove the model to predict an outcome of survival at 1 year, while a positive value drove an outcome of death. Yellow and purple colors represent low and high numerical values of the feature in the dataset, respectively. For example, a higher age at listing tended to drive the model to predict death, since there are increasingly purple dots as the SHAP value increases.

We have plotted the values of the top 7 features which contributed most to predicting mortality via the Shapley Additive Explanations (SHAP) method (Figure 3). SHAP analysis allows us to examine the importance of each feature in relationship each other in the full model. The most salient features which explained 1-year mortality were recipient factors: functional status at transplant, age, pulmonary capillary wedge pressure (PCWP), cardiac output (CO), ECMO usage, and serum creatinine. In Figure 3, we can appreciate that a worse functional status (more purple color) increases the prediction of mortality (more positive SHAP value on the *x*-axis). Similarly, increasing age at listing and transplant also increases mortality, as does ECMO usage and higher creatinine. For all patients in this cohort, an age at transplant of 53 years or under was protective in the model, while an age of 54 or older was predictive of mortality (Figure S3). Interestingly, higher PCWP and lower cardiac output were protective against mortality. Although the top 7 most important variables were recipient characteristics, donor factors also made important contributions to the model. For example, higher donor PaO_2_ was consistently protective for all patients.

**Figure 4:**
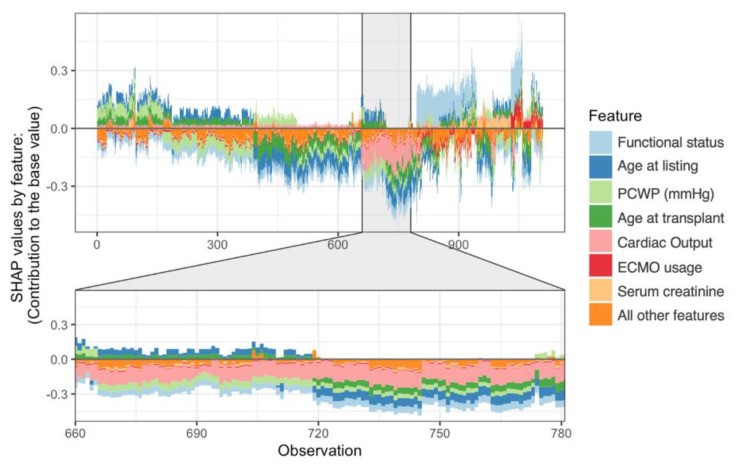
SHAP force plot of the top 7 most important features, by mean SHAP value, with all other features grouped together (orange bars). The *x*-axis represents each patient in the development cohort. This plot contains information about how features contributed to model prediction for each observation. Bars further away from a SHAP value of zero, in either positive or negative direction, mean that the feature contributed more to the model. Features with negative SHAP values predicted survival while positive values predicted death. This figure does not illustrate the actual values and directionality of the features; please refer to Figure 3.

We can also observe the importance of the top 7 variables (with all other variables treated as a single group) on predicting mortality for each individual (Figure 4). There is significant heterogeneity within this cohort as there are distinct clusters of patients (“observations” on the *x*-axis) in terms of how features vary in driving a dead (positive SHAP value) vs. alive (negative SHAP value) prediction. For example, for observations 660–780, cardiac output played a more important role in the XGBoost model than it did for observations 800–900, since the pink bars (cardiac output) had a larger absolute SHAP value. Similarly, functional status was a more important predictor for observations 820–920 compared to 660–780. There were no statistically significant associations between the observed clusters and support device type.

## 4. Discussion

First, we have built a machine learning model which is the first of its kind to predict 1-year post-transplant mortality in patients bridged with tMCS. Second, our model was able to accurately predict mortality using only pre-transplant variables and thus provides clinically relevant information for pre-operative risk stratification. Third, we have presented a framework for ML model interpretation, which has allowed us to examine the most clinically meaningful variables and their influences on driving the model’s prediction of mortality.

Many risk scores have been developed for heart transplant recipients and generally exhibit wide variations and poor-to-modest performance depending on the specific validation cohort [17]. Like the well-established Society of Thoracic Surgeons (STS) Adult Cardiac Surgery Models [18], risk scores predominantly use standard regression techniques and may not fully capture complex and non-linear interactions between variables. Notably, the IMPACT score, one of the more well-studied heart transplant risk tools, typically performs with an AUC in the 0.5 s to 0.6 s. To overcome this obstacle of standard regression, various machine learning algorithms have also been built for various topics within cardiac surgery. XGBoost and random forest techniques have been used to predict operative mortality for cardiac surgery operations without STS risk scores [19]. XGBoost models for predicting various outcomes following surgical aortic valve replacement have also been shown to calibrate well with models derived from STS risk scores [20], and others have offered marginal survival prediction improvements for the major STS operations with risk scores [21].

ML approaches have also demonstrated their potential within the field of transplantation. Kampaktsis et al. [12] built various ML algorithms using a general adult OHT population from the UNOS dataset and achieved a best AUC of 0.689. Some of the most salient features identified in their best performing model, which used the AdaBoost framework [22], are the same as ones from our XGBoost model, including recipient creatinine and age. However, our model also identified new variables including the hemodynamic variables of pulmonary capillary wedge pressure and cardiac output. Yoon et al. [23] also attempted post-transplant mortality predictions in a similar UNOS cohort by using Tree of Predictors, a novel and complex ensemble method, however, their 1 year AUC was 0.641. Additionally, deep learning frameworks have shown success in predicting post-transplant hospitalization in pediatric kidney, liver, and heart transplantations [24].

Model interpretability remains a challenge for machine learning. Many ML methods are considered “black boxes” since they rely on sophisticated back-end processes which are often incomprehensible to humans [9,25]. Consequently, clinicians, who must have concrete, clinically relevant, and actionable data, might prefer traditional statistical methodologies like regression-based techniques since the contribution of individual variables can be examined. In this study, we have presented a novel implementation of Shapley Additive Explanations on the UNOS dataset, which opens an otherwise black box. We prefer SHAP analysis to other model-agnostic methods since it allows for convenient analysis of individual features on a patient level [15].

The features identified by SHAP analysis, integrating both Figure 3 and Figure 4, are critical in understanding the tMCS bridge to transplant population. Many of the explanations are clinically intuitive. Worse functional status, greater age, ECMO usage, and higher serum creatinine are all classic risk factors and are expected to increase mortality. Functional status is a mandated field in the UNOS database and uses the Karnofsky performance score, with 10 possible values ranging from 100% (healthy, no symptoms) to 10% (moribund) in 10% increments [26]. The score is subjective by nature and not calculated from any laboratory values, but in our case, shows high utility in predicting mortality with lower scores predicting death.

However, having a lower PCWP was predictive of mortality, while a lower CO tended to be protective. This novel and paradoxical finding may reflect poor baseline health in patients requiring more intensive hemodynamic support, which would drive CO up and PCWP down. Although SHAP analysis attempts to visually explain individual feature influence in a way easily digestible for people, it is important to remember that the final XGBoost model analyzes complex, non-linear relationships between all variables simultaneously. Thus, explanations are most relevant when looking at the model holistically and at general population-level trends. Additionally, the significant heterogeneity within our cohort is interesting. Although different tMCS support devices have different clinical indications, risk factors, and post-transplant outcomes [8], device type alone did not explain patient clustering in our cohort. This observation further highlights the importance of using sophisticated methods like XGBoost to identify high-dimensional relationships which would otherwise be missed.

### Limitations

There was class imbalance for the primary outcome (more than 7 survivors for each non-survivor), which generally limits the performance of any classification model [27]. Compared to more general OHT cohorts, the tMCS cohort is relatively small, especially since we restricted our time window to 2009 through 2017. We recognize that the 2018 UNOS policy change has resulted in practice changes and outcomes particularly for those on mechanical support; however, given concerns regarding an inappropriate rise in tMCS utilization post-policy change and potential for “gaming the system” [28,29], we felt that restricting our study to a pre-policy change cohort would better allow us to evaluate true patient-level factors without the noise of provider- and center-level variability. Nonetheless, the lack of validation in the most contemporary post-policy change cohort remains a limitation. Additionally, given the continuous developments in tMCS device innovation, our model may not be applicable for future tMCS populations. We also only examined 1-year mortality since this is often used as a quality metric and allows more equipoise comparison to other risk scores which use the same outcome. Longer survival time windows, as well as other post-operative outcomes such as renal failure, rehospitalization, and graft failure, should be explored. Finally, as with any model, our results should be validated with external datasets, though this is notably difficult to achieve given the UNOS database’s comprehensive coverage of all transplants in the United States.

## 5. Conclusions

An interpretable machine learning model trained on a large clinical database demonstrated leading performance in predicting 1-year mortality for patients bridged to heart transplantation with tMCS. We have delineated a machine learning framework which may be used to investigate individual factors in pre-operative risk assessment for populations where existing risk scores are poorly validated.

## Figures and Tables

**Table 1:** Baseline characteristics and demographics for patients who required temporary mechanical circulatory support for bridge to transplant between survivors and non-survivors at 1-year post-transplant. Variables represent recipient characteristics unless otherwise indicated.

Variable	Survived *n* = 1405	Died *n* = 179	*p* Value
Age, years	55 (45–62)	59 (50–64)	<0.001
Male sex	1037 (73.8%)	132 (73.7%)	0.99
Diabetes	383 (27.3%)	65 (36.3%)	0.01
Body mass index (kg/m^2^)	26.0 (23.1–29.8)	27.2 (23.7–30.8)	0.019
Ischemic time, hours	3.1 (2.4–3.8)	3.2 (2.6–3.8)	0.42
Total days on waitlist	34 (12–90)	43 (12–117)	0.17
Ethnicity			0.34
White	903 (64.3%)	111 (62.0%)	
Black	333 (23.7%)	38 (21.2%)	
Hispanic	106 (7.5%)	19 (10.6%)	
Other	63 (4.5%)	11 (6.1%)	
Donor age, years	30 (23–41)	34 (23–45)	0.12
Donor male sex	949 (67.5%)	117 (65.4%)	0.56
Hemodynamics at listing			
Cardiac output	3.9 (3.14–4.82)	4 (3.185–4.7)	0.70
PCWP	22.0 (16.0–28.0)	21.0 (15.0–28.0)	0.66
MPAP	32 (25–38)	31.5 (24–39.5)	0.45
PA systolic pressure	45 (36–54)	48 (37–56.5)	0.17
PA diastolic pressure	23 (17–28)	22 (16.5–29.5)	0.99
Inotrope usage	661 (47.0%)	82 (45.8%)	0.75
Hemodynamics at transplant			
Cardiac output	4 (3.19–5)	4.14 (3.4–5.1)	0.10
PCWP	22.0 (16.0–28.0)	20.5 (13.5–27.0)	0.04
MPAP	31 (24–39)	30 (23–38)	0.28
PA systolic pressure	45 (35–55)	42 (34–55)	0.36
PA diastolic pressure	23 (16–29)	22 (16–28)	0.22
Inotrope usage	813 (57.9%)	89 (49.7%)	0.04
Serum creatinine (mg/dl)	1.14 (0.90–1.50)	1.30 (1.00–1.70)	0.001
Total bilirubin (mg/dL)	0.9 (0.6–1.4)	1.0 (0.6–1.7)	0.05
Implantable cardiac defibrillator	1015 (72.6%)	142 (80.2%)	0.03

PCWP: pulmonary capillary wedge pressure; MPAP: mean pulmonary artery pressure; PA: pulmonary artery. Data are presented as median (IQR) for continuous measures, and *n* (%) for categorical measures.

## Data Availability

Access to the United Network for Organ Sharing/Organ Procurement and Transplantation Network Registry can be requested at https://optn.transplant.hrsa.gov/data/ (accessed on 25 August 2022).

## Supplementary Materials

The following supporting information can be downloaded at: https://www.mdpi.com/article/10.3390/jcdd9090311/s1, Figure S1: Unadjusted one year post-transplant Kaplan-Meier survival curves for those bridged to transplant with temporary mechanical circulatory support (tMCS BTT) and those without (No tMCS), compared using the log-rank test; Figure S2: Precision recall curve for the XGBoost model. The color legend on the right represents a certain pair of sensitivity and false positive rate for each point on the curve; Table S1: Baseline characteristics and demographics for patients who required temporary mechanical circulatory support for bridge to transplant (tMCS BTT) and those who did not. Variables represent recipient characteristics unless otherwise indicated.; Table S2: Feature (i.e., variable) importance ranked by gain (i.e., predictive utility in the final model). Features represent recipient characteristics unless otherwise indicated. Descriptions are derived from the thoracic organ Standard Transplant Analysis and Research (STAR) files.

## Abbreviations

AUC	Area under the Receiver Operator Characteristics Curve
BTT	bridge to transplant
CO	cardiac output
ECMO	extracorporeal membrane oxygenation
IABP	intra-aortic balloon pump
ML	machine learning
OHT	orthotopic heart transplantation
PCWP	pulmonary capillary wedge pressure
PVAD	percutaneous ventricular assist device
SHAP	Shapley Additive Explanations
STS	Society of Thoracic Surgeons
tMCS	temporary mechanical circulatory support
UNOS	United Network for Organ Sharing
