# Supplementary material for: Pre-operative Machine Learning for Heart Transplant Patients Bridged with Temporary Mechanical Circulatory Support
Source: J Cardiovasc Dev Dis. 2022 Sep 19;9(9):311. doi: 10.3390/jcdd9090311 (PMC9500687; doi:10.3390/jcdd9090311)

Table S1. Baseline characteristics and demographics for patients who required temporary mechanical circulatory support for bridge to transplant (tMCS BTT) and those who did not. Variables represent recipient characteristics unless otherwise indicated.

	No tMCS n=17,433	tMCS BTT n=1,584	P value
Age, years	57 (47-63)	56 (46-62)	<0.001
Male sex	12,985 (74.5%)	1,169 (73.8%)	0.55
Diabetes	4,994 (28.6%)	448 (28.3%)	0.76
Body mass index (kg/m^2)	27.1 (23.8-30.7)	26.1 (23.2-30.0)	<0.001
Ischemic time, hours	3.1 (2.4-3.8)	3.1 (2.4-3.8)	0.81
Total Days on Waitlist	116 (35-305)	34 (12-92)	<0.001
Follow-up Time, years	5.0 (3.0-7.7)	4.1 (2.9-6.9)	<0.001
Ethnicity			0.35
White	11,503 (66.0%)	1,014 (64.0%)	
Black	3,753 (21.5%)	371 (23.4%)	
Hispanic	1,381 (7.9%)	125 (7.9%)	
Other	796 (4.6%)	74 (4.7%)	
Donor Age, years	30 (22-41)	31 (23-41)	0.28
Donor Male Sex	12,315 (70.6%)	1,066 (67.3%)	0.005
Hemodynamics at Listing			
Cardiac Output	4.16 (3.35-5.03)	3.9 (3.16-4.8)	<0.001
PCWP	19.0 (13.0-25.0)	22.0 (16.0-28.0)	<0.001
MPAP	29 (22-36)	32 (25-38)	<0.001
PA Systolic Pressure	42 (32-53)	45 (36-55)	<0.001
PA Diastolic Pressure	20 (15-26)	23 (17-28)	<0.001
Inotrope usage	5,672 (32.5%)	743 (46.9%)	<0.001
Hemodynamics at Transplant			
Cardiac Output	4.42 (3.6-5.4)	4 (3.2-5)	<0.001
PCWP	17.0 (11.0-24.0)	22.0 (15.0-28.0)	<0.001
MPAP	26 (20-34)	31 (24-39)	<0.001
PA Systolic Pressure	38 (30-49)	45 (35-55)	<0.001
PA Diastolic Pressure	18 (13-24)	23 (16-29)	<0.001
Inotrope usage	6,205 (35.6%)	902 (56.9%)	<0.001
Serum creatinine (mg/dl)	1.20 (0.95-1.50)	1.19 (0.90-1.50)	0.16
Total bilirubin (mg/dL)	0.7 (0.5-1.1)	0.9 (0.6-1.5)	<0.001
Implantable cardiac defibrillator	13,824 (79.9%)	1,157 (73.4%)	<0.001

PCWP: pulmonary capillary wedge pressure; MPAP: mean pulmonary artery pressure; PA: pulmonary artery. Data are presented as median (IQR) for continuous measures, and n (%) for categorical measures.

Table S2. Feature (i.e., variable) importance ranked by gain (i.e., predictive utility in the final model). Features represent recipient characteristics unless otherwise indicated. Descriptions are derived from the thoracic organ Standard Transplant Analysis and Research (STAR) files.

Rank	Feature	Description	Gain
1	FUNC_STAT_TRR	Functional Status (Karnofsky performance score)	0.158512516
2	INIT_AGE	Age in years at time of listing	0.107789265
3	HEMO_PCW_TRR	Pulmonary capillary wedge pressure at transplant	0.098986965
4	HEMO_CO_TRR	Cardiac output at transplant	0.084797351
5	AGE	Age at transplant	0.079323797
6	CREAT_TRR	Serum creatinine at transplant	0.05437153
7	ECMO_TRR	ECMO support at transplant	0.049576131
8	SGOT_DON	Donor last aspartate aminotransferase	0.022711094
9	HEMO_CO_TCR	Cardiac output at listing	0.022331076
10	PRIOR_CARD_SURG_TYPE_TRR13	Surgical ventricular remodeling surgery between listing and transplant	0.021407742
11	BMI_CALC	Body mass index	0.021316674
12	TBILI	Serum total bilirubin at transplant	0.020194779
13	PRIOR_CARD_SURG_TYPE_TCR16	Surgical ventricular remodeling surgery before listing	0.018108468
14	HEMO_PCW_TCR	Pulmonary capillary wedge pressure at listing	0.017537055
15	INIT_WGT_KG_CALC	Weight at listing	0.017062931
16	DAYSWAIT_CHRON	Total days on waiting list	0.016572684
17	HEMO_SYS_TCR	Systolic pressure at listing	0.011443448
18	HGT_CM_CALC	Height at listing	0.011385361
19	HEMO_PA_MN_TCR	Mean pulmonary artery pressure at listing	0.010553371
20	AGE_DON	Donor age	0.008990021
21	PRIOR_CARD_SURG_TYPE_TRR6	Coronary artery bypass graft + valve repair/replacement between listing and transplant	0.008841328
22	PO2_FIO2_DON	Donor FiO_2 (if applicable)	0.008775327
23	PO2	Donor pO_2 on 100% FiO_2	0.008391518
24	CPRA	Calculated panel reactive antibodies	0.007533868
25	EDUCATION3	Attended college/technical school	0.006110179
26	SGPT_DON	Donor last alanine aminotransferase	0.005660703
27	HEMO_PA_DIA_TCR	Diastolic pulmonary artery pressure at listing	0.005061204
28	TRANSFUS_TERM_DON	Donor number of transfusions during terminal hospitalization	0.004492959
29	TCR_DGN8	Viral dilated cardiomyopathy diagnosis	0.004488877
30	BMI_TCR	Body mass index at listing	0.003839232
31	TCR_DUR_ABSTAIN	If prior smoker, duration of abstinence	0.003825491
32	END_HGT_CM_CALC	Height at transplant	0.003633827

33	HEMATOCRIT_DON	Donor hematocrit	0.003621744
34	BMI_DON_CALC	Donor body mass index	0.003540091
35	ABO3	AB blood type	0.003346749
36	HEMO_PA_DIA_TRR	Diastolic pulmonary artery pressure at transplant	0.003235971
37	ETHNICITY	Hispanic ethnicity	0.003149958
38	CORONARY_ANGIO	Abnormal donor coronary angiogram	0.003001513
39	CREAT_DON	Donor creatinine	0.00277229
40	ABO0	Type O blood	0.00255622
41	BUN_DON	Donor blood urea nitrogen	0.00242364
42	IABP_TCR	Intraaortic balloon pump support at listing	0.002228423
43	DAYS_STAT1A	Days in Status 1A	0.001749797

Figure S1. Unadjusted one year post-transplant Kaplan-Meier survival curves for those bridged to transplant with temporary mechanical circulatory support (tMCS BTT) and those without (No tMCS), compared using the log-rank test.

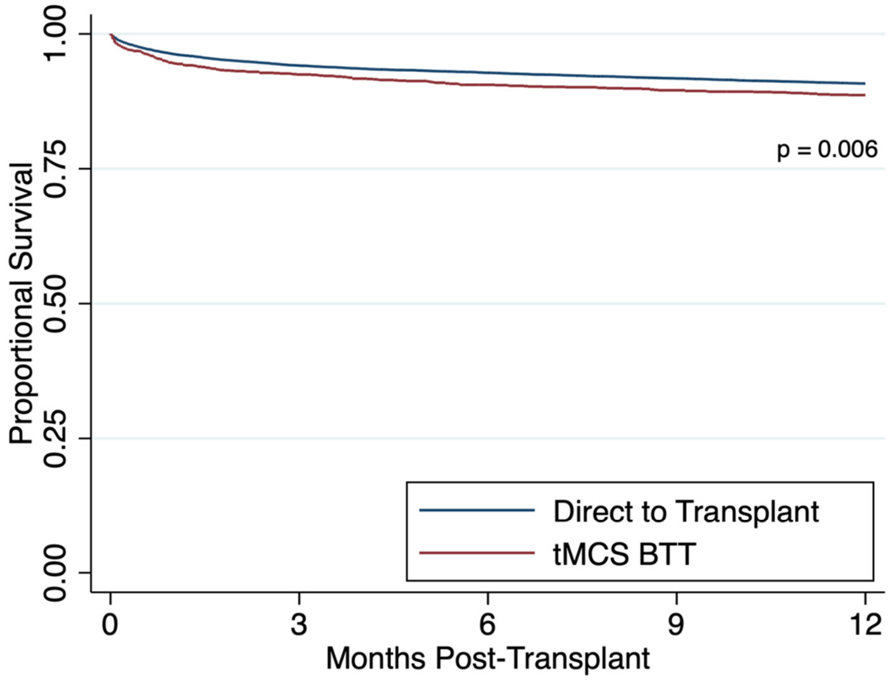

Number at risk

	0	3	6	9	12
No tMCS	17433	16368	16125	15931	15742
tMCS BTT	1584	1460	1426	1409	1393

Figure S2. Precision recall curve for the XGBoost model. The color legend on the right represents a certain pair of sensitivity and false positive rate for each point on the curve.

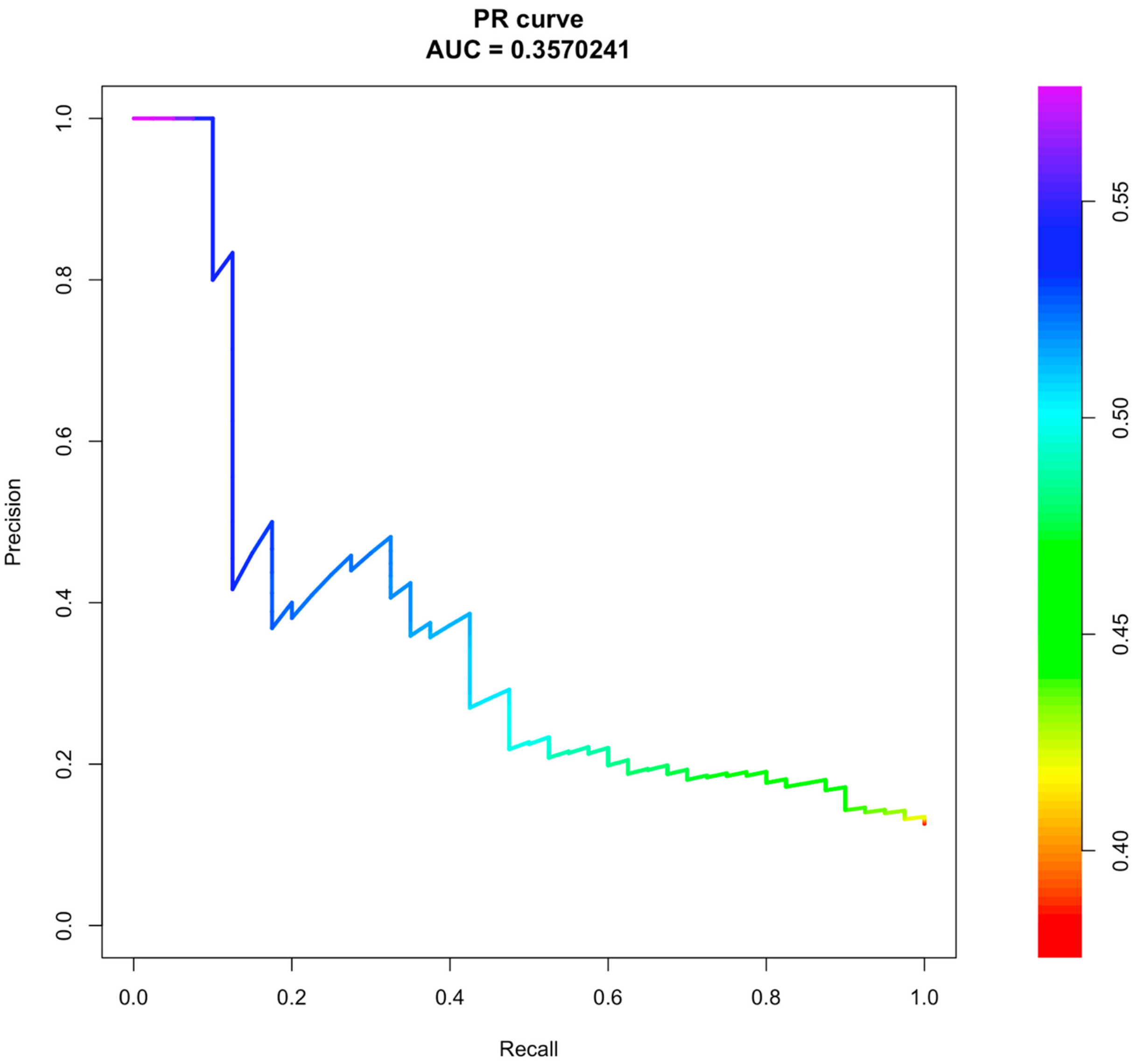

Figure S3. SHAP dependence plot for actual age vs. age SHAP value. A clear threshold is observed between ages 53 and 54, above which the model predicted death (above 0.00 on y-axis) and below which the model predicted survival (under 0.00 on y-axis).

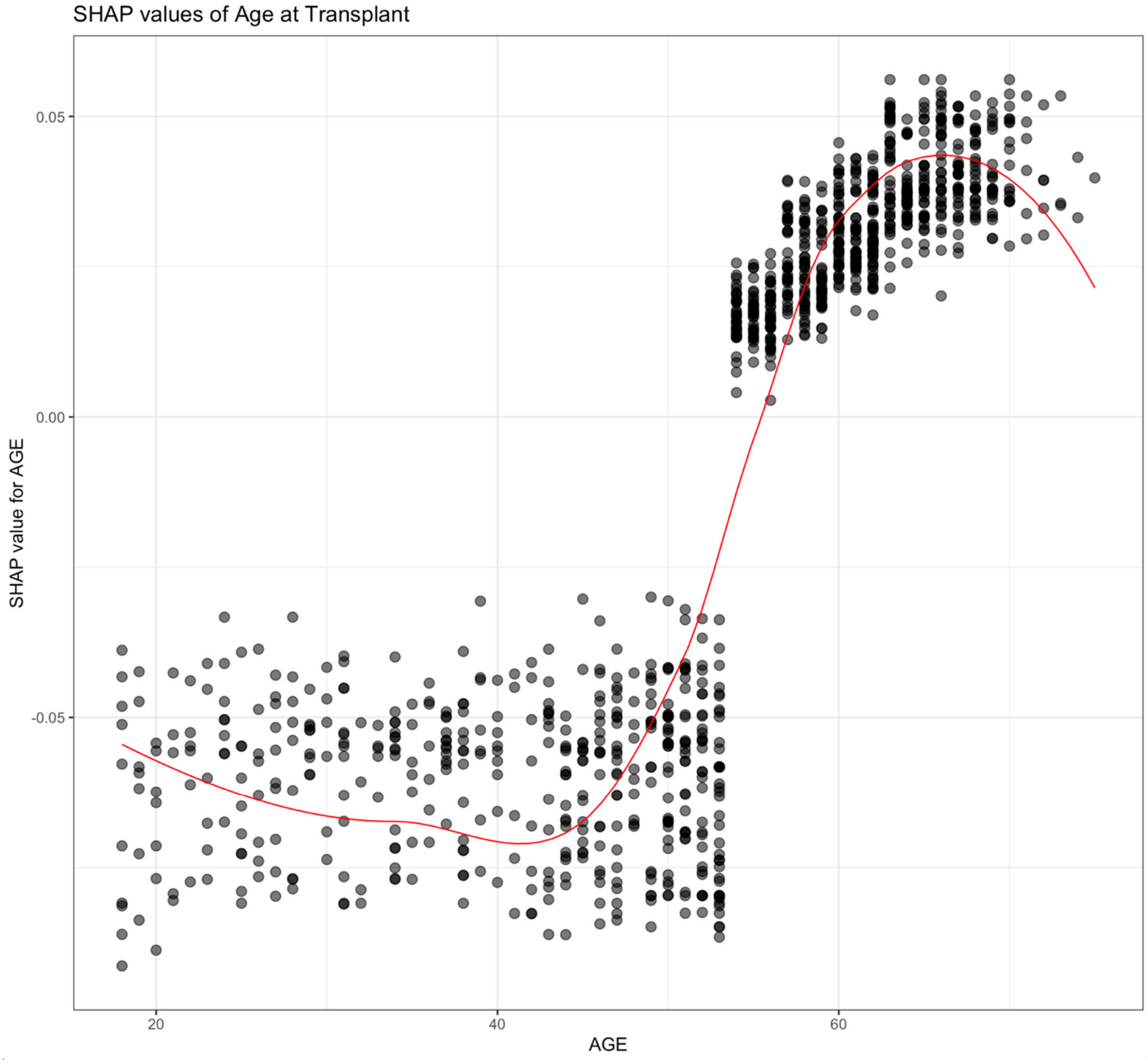

Supplement: Supplementary file 1 [file jcdd-09-00311-s001.zip › jcdd-1911034-supplementary.pdf]
